# Impact of Adrenalectomy on Morbidity in Patients with Non-Functioning Adrenal Cortical Tumours, Mild Hypercortisolism and Cushing’s Syndrome as Assessed by National and Quality Registries

**DOI:** 10.1007/s00268-021-06214-0

**Published:** 2021-06-27

**Authors:** Lo Hallin Thompson, Jonas Ranstam, Martin Almquist, Erik Nordenström, Anders Bergenfelz

**Affiliations:** 1 Department of Surgery, Skåne University Hospital, 22185 Lund, Sweden; 2 Department of Clinical Sciences, Lund University, Lund, Sweden; 3 Department of Orthopaedics, Skåne University Hospital, 22185 Lund, Sweden

## Abstract

**Background:**

The impact of adrenalectomy on morbidity in patients with mild hypercortisolism and non-functioning adrenocortical adenoma is unclear. The present study evaluated morbidity before and after adrenalectomy in patients with benign adrenocortical tumour with Cushing´s syndrome (CS), autonomous cortisol secretion (ACS) and non-functioning adrenocortical adenoma as assessed by national and quality registries.

**Methods:**

Patients registered in the Scandinavian Quality Register for Thyroid, Parathyroid and Adrenal Surgery (SQRTPA) 2009–2017 with CS, ACS or non-functioning adrenocortical adenoma, were included in this retrospective study and analysed with age- and sex-matched controls, 1:3. Morbidity associated with CS was assessed pre- and postoperatively by analysing data from the Swedish National Patient Register and the Swedish Prescribed Drug Register.

**Results:**

Some 271 patients were included, CS (127), ACS (45) and non-functioning adrenocortical adenoma (99), with 813 matched controls. The frequency of hypertension was almost 50% in all tumour groups. Antihypertensive medication preoperatively was more frequent in all tumour groups compared with controls. No preoperative differences in medication were detected between patients with CS and ACS. A decrease in the use of hypertensive drugs was noticed annually for all patient groups after adrenalectomy.

**Conclusions:**

Hypertension is common in patients with benign adrenocortical tumours regardless of cortisol hypersecretion. The use of antihypertensive drugs in patients with CS, ACS and non-functioning adrenocortical adenoma was reduced after adrenalectomy. These findings highlight the need for a randomized controlled trial to investigate the impact of adrenalectomy on morbidity in patients with mild hypercortisolism.

**Supplementary Information:**

The online version contains supplementary material available at 10.1007/s00268-021-06214-0.

## Introduction

Adrenal incidentalomas are frequently detected in routine imaging with a prevalence of 1–4 percent [1, 2]. The majority of these tumours are benign cortical adenomas not associated with clinically relevant hormonal hypersecretion [3, 4]. In 5–30 percent of incidentalomas there is mild hypersecretion of cortisol without classical signs of Cushing´s syndrome (CS), also referred to as autonomous cortisol secretion (ACS) [5–7]. Patients with ACS exhibit some features similar to Cushing´s syndrome with higher risk of glucose intolerance, hypertension, hyperlipidaemia and osteoporosis [8–11]. Recent reports show that adrenalectomy may ameliorate cardiovascular risk factors and decrease the risk for fracture [9, 12–15]. The European Society of Endocrinology recommends that patients with ACS should be screened for hypertension, type-2 diabetes mellitus and asymptomatic vertebral fractures [16].

It is still unclear if patients with ACS benefit from surgical treatment, comprehensive patient series are absent [9, 14, 17]. Benign adrenal tumours without any detectable hormonal hypersecretion are referred to as non-functioning adrenocortical tumours. However, it has been reported that these patients have an increased risk of diabetes mellitus and hypertension [18–21]. Some studies have suggested that patients with non-functioning adrenocortical adenoma have a minor hypersecretion of cortisol, albeit difficult to detect with current biochemical methods [19, 22]. However, no large studies have investigated whether patients with non-functioning adrenocortical adenomas benefit from surgery with regards to cardiovascular risk factors.

The aim of this study was to evaluate the impact of adrenalectomy on morbidity potentially related to cortisol excess, in patients with CS, ACS and non-functioning adrenocortical adenoma, as assessed by national and quality registries, with a special focus on cardiovascular disease and related risk factors.

## Material and methods

### Data sources

The Scandinavian Quality Register for Thyroid, Parathyroid and Adrenal Surgery (SQRTPA) was established in 2004 and is recognized by the Swedish National Board of Health and Welfare as the national register within the field [23]. The Swedish Endocrine Surgical Society recommends participation. Adrenalectomy is currently performed at seven university hospitals that all participate. Surgeons or specialized nurses perform registration. Data is audited annually. Coverage is assessed relating the proportion of reported patients to patients registered in The Swedish National Patient Register (NPR).

The National Board of Health and Welfare administrate national registers to facilitate analysis and development of Swedish healthcare [24]. Quality and validity checks of data are performed regularly. As of 1987, the NPR includes all in-patient care at all hospitals in Sweden, including discharge diagnoses and surgical procedures. From 2005 the Swedish Prescribed Drug Register (SPDR) contains information about drugs prescribed and dispensed in Sweden, including Anatomical Therapeutic Chemical classification system (ATC) codes, drug doses, dates and the prescriber’s profession and practice. Registration is mandatory for all pharmacies in Sweden.

### Patients

Patients registered as undergoing adrenalectomy 2009–2017 in SQRTPA, with CS, ACS or non-functioning adrenocortical adenoma, were included in the study. Patients were identified by preoperative laboratory findings and histopathology (adrenocortical adenoma or adrenocortical hyperplasia). CS and ACS was diagnosed by a specialist in endocrinology and based on applicable criteria at the time of diagnosis with 1-mg-overnight dexamethasone-suppression (DST) for initial evaluation [16, 25]. Non-functioning adrenocortical adenoma were considered when hormonal evaluation was normal. Preoperative data on age, sex, body mass index (BMI), blood pressure, associated diseases, tumour detection, indication for adrenalectomy, tumour size, tumour side, surgical technique, postoperative length of stay were retrieved from SQRTPA. Postoperative adrenal insufficiency was defined as substitution with cortisone. The first follow-up was performed approximately four to six weeks postoperatively. Hypertension was defined as a measured blood pressure > 140/90 mmHg or treatment with antihypertensive drugs.

Morbidity in patients with CS, ACS and non-functioning adrenocortical adenoma were compared to controls before and after adrenalectomy.

Data on drug prescription and inpatient diagnoses were retrieved from SPDR and NPR respectively. Data was extracted on December 28^th^, 2018. Drugs used for treatment of hypertension, diabetes mellitus, hyperlipidaemia, osteoporosis, infectious diseases and depressive disorders were identified through ATC-codes (Table 1). Defined daily dose (DDD) was used to calculate drug consumption per patient-year, based on dispensed prescriptions the year before adrenalectomy and annually after adrenalectomy. Patients with no registered medication were set to zero. The ICD-10-SE (International Statistical Classification of Diseases and Related Health Problems-Tenth Revision-Sweden) was used to evaluate morbidity pre- and postoperatively. Diagnoses were divided in disease groups; hypertension, ischemic heart disease, atherosclerosis, diabetes mellitus, hyperlipidaemia and osteoporosis (Table 1). Inpatient diagnoses registered from 1999 until adrenalectomy were used for preoperative evaluation and until register extract postoperatively. Diagnoses and dispensed prescriptions at the time for surgery were considered as preoperative data.

**Table 1:** ATC-codes and ICD-10-SE

	ATC-codes
Hypertension	C02, C03, C07, C08, C09
Diabetes type 2	A10A, A10B
Hyperlipidaemia	C10
Osteoporosis	M05BA, M05BB, M05BX, H05AA02, G03XC01
Infectious diseases	J01
Depression	N06A
	ICD-10-SE
Hypertension	I10-I15
Ischemic heart disease	I20-I25
Atherosclerosis	I70
Diabetes type 2	E11
Hyperlipidaemia	E78
Osteoporosis	M80- M82

A control group with three controls per patient, matched by age and sex were identified by Statistics Sweden (the government agency for population statistics) through the Swedish Population register [26]. The controls were used as referents for dispensed prescriptions and comorbidity. All controls were assigned the date of surgery of their corresponding case as reference date.

### Statistical analysis

Aggregated data are presented as frequencies, medians with interquartile range (IQR) or with mean and standard deviation (SD). Frequencies were assessed based on provided data, no data are excluded or imputed. Statistical hypothesis tests were performed using the Chi-squared test for categorical variables and either Student´s *t* test or Wilcoxon´s Rank-Sum test for continuous variables. The pre- and postoperative incidence density rates of disease diagnoses are calculated as the sum of diagnoses (ICD-10) in each disease group relative to the amount of person time at risk from start of follow up until surgery and from surgery until end of follow up respectively. The confidence intervals for the incidence density rate ratios (IRR) were calculated under the assumption of a Poisson distribution. All analyses were conducted using Stata v16.1 (Stata Corp. 2019, Stata Statistical Software: Release 16. College Station, TX: StataCorp LLC). *p* values below 0.05 were considered statistically significant.

The ethics committee, Lund university approved the study (No. 2017/800).

## Results

**Fig. 1:**
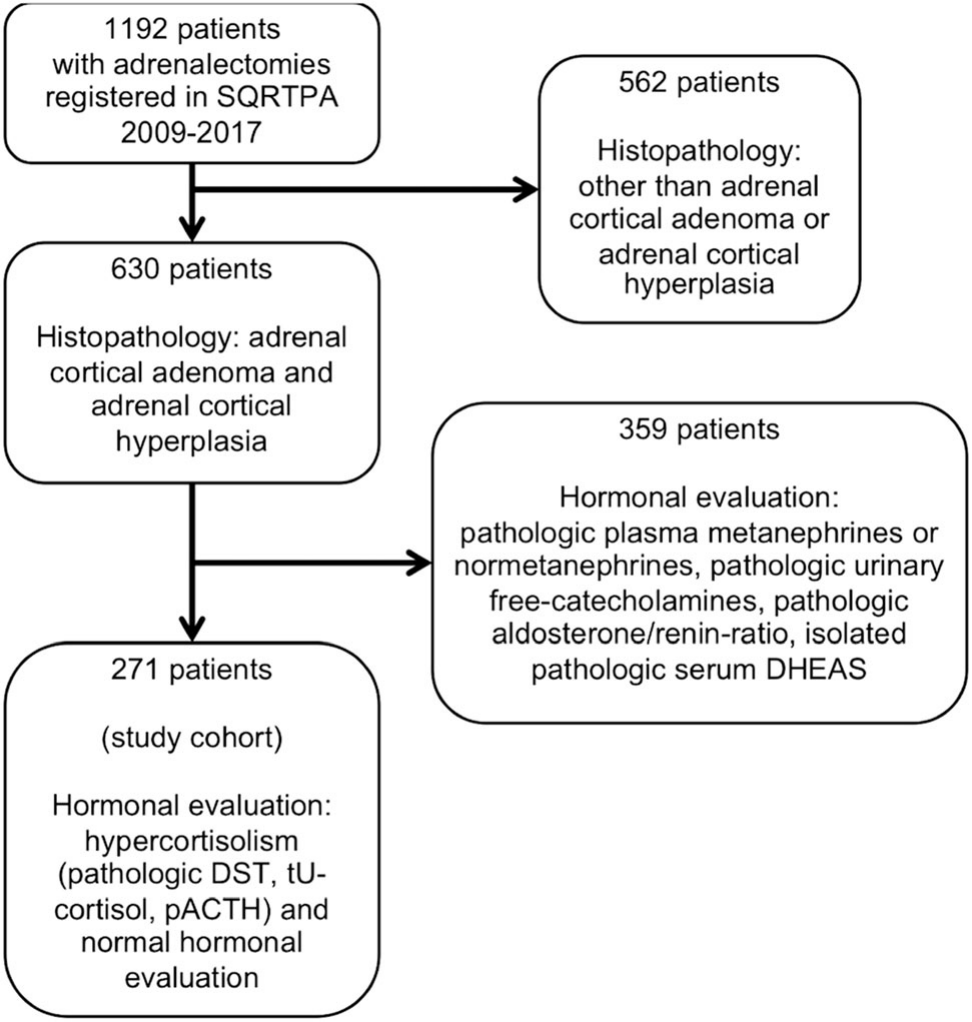
Flowchart of cohort creation. *DST* dexamethasone suppression test, *ACTH* adrenocorticotropic hormone, *DHEAS* dehydroepiandrosteronesulfate

Some 271 patients undergoing adrenalectomy were included in the study (Fig. 1). There were 127 patients with CS, 104 (81.9 percent) women, mean (SD) age 57 (13) years, 45 patients with ACS, 31 (68.9 percent) women, mean age 65 (10) years, 99 patients with non-functioning adrenal cortical adenoma, 59 (59.6 percent) women, mean age 61 (12) years and 813 controls.

Pre- and perioperative characteristics and surgical outcome are summarized in Table 2. Almost 50 percent of the patients had hypertension at the time for surgery. Patients with CS were more often detected by adrenal related symptoms compared with the other patient groups. Postoperative adrenal insufficiency was registered in SQRTPA more frequently in patients with CS and ACS compared with patients with non-functioning adrenocortical adenoma at discharge and at first follow-up.

**Table 2:** Patient characteristics at the time of surgery and surgical outcome registered in the Scandinavian Quality Register for Thyroid Parathyroid and Adrenal Surgery

	CS *n* = 127	ACS *n* = 45	NFAA *n* = 99
Sex (female), *n* (%)	104 (81.9)	31 (68.9)	59 (59.6)
Age (years), mean (SD)	56.9 ± 12.6	65.0 ± 10.4	60.5 ± 12.1
Hypertension, *n* (%)	84 (66.1)	29 (64.4)	49 (49.5)
Diabetes, *n* (%)	24 (18.9)	6 (13.3)	14 (14.1)
Osteoporosis, *n* (%)	18 (14.2)	8 (17.8)	3 (3.0)
BMI (kg/kvm), mean (SD)	26.2 ± 9.5	26.0 ± 8.6	25.0 ± 9.6
Incidentaloma, *n* (%)	72 (56.7)	43 (95.6)	85 (85.9)
*Indication for surgery, n (%)*			
Hypercortisolism	127 (100)	45 (100)	0
Tumour size > 40 mm	2 (1.6)	5 (1.1)	20 (20.0)
Suspected malignancy on CT	25 (19.7)	18 (40.0)	38 (30.4)
Suspected metastasis	0	1 (2.2)	2 (2.0)
Bilateral tumours, *n* (%)	19 (15.0)	4 (8.9)	4 (4.0)
Proportion hyperplasia	17/19	1/4	3/4
Tumour size (mm), mean (SD)^a^	33 ± 14	37 ± 11	37 ± 15
*Surgical technique, n (%)*			
Open	7/126 (5.6)	4 (8.9)	14/98 (14.3)
Endoscopic	119/126 (94.4)	41 (91.1)	84/98 (85.7)
LOS (days), median (IQR)^b^	3 (2–5)	3 (2–3)	2 (2–4)
*Histopathology, n (%)*			
Adrenal cortical adenoma	94 (74.0)	41 (91.1)	90 (90.9)
Adrenal cortical hyperplasia	33 (26.0)	4 (8.9)	9 (9.1)
Postoperative treatment due to adrenal insufficiency: postoperatively, *n* (%)	91/125 (72.8)	25/42 (59.5)	10/95 (10.5)
Postoperative treatment due to adrenal insufficiency: first follow up, *n* (%)	87/121 (71.9)	22/44 (50.0)	14/97 (14.4)

*CS* Cushing’s syndrome, *ACS* Autonomous cortisol secretion, *NFAA* Non-functioning adrenocortical adenoma, *BMI* Body mass index, *LOS* Postoperative length of hospital stay, *SD* Standard deviation, *IQR* Inter quartile range

^a^ CS *n* = 123, ACS *n* = 44, NFAA *n* = 96

^b^ CS *n* = 126, ACS *n* = 44, NFAA *n* = 97

Morbidity evaluated by medication in the different disease groups preoperatively compared to controls (mean DDD/patient/year) is shown in Table 3. Antihypertensive medication was more frequently used in all tumour groups compared with the control group. No statistical significant differences in medication between patients with CS and ACS could be detected. Patients with CS and ACS had a higher frequency of dispensed medications than the control group, in all ATC-groups, except for antibiotics and antidiabetics. Patients with non-functioning adrenocortical adenoma medicated more often for hypertension, diabetes and hyperlipidaemia than the control group. No statistical differences were detected in the use of antibiotics among the patient groups and controls.

**Table 3:** Defined daily dose (DDD) per patient per year, one year before adrenalectomy in the patients and controls and grouped according to anatomical therapeutic chemical (ATC) codes. Mean and standard deviation is shown

	CS *n* = 127	ACS *n* = 45	NFAA *n* = 99	Control group *n* = 813	CS vs. ACS *p* value	CS vs. NFAA *p* value	CS vs. Control group *p* value	ACS vs. NFAA *p* value	ACS vs. Control group *p* value	NFAA vs. Control group *p* value
Hypertension	572.3 (928.0)	465.5 (719.6)	345.1 (495.6)	236.1 (477.3)	0.484	**0.029**	** < 0.001**	0.246	**0.002**	**0.033**
Diabetes	73.5 (197.8)	109.1 (310.2)	91.2 (320.1)	39.6 (201.7)	0.379	0.611	0.077	0.755	**0.030**	**0.026**
Hyperlipidaemia	152.3 (378.6)	133.0 (257.0)	114.1 (269.3)	64.8 (212.4)	0.752	0.397	** < 0.001**	0.693	**0.038**	**0.035**
Osteoporosis	35.5 (120.2)	39.2 (102.3)	6.8 (39.2)	7.6 (56.6)	0.854	**0.023**	** < 0.001**	**0.007**	** < 0.001**	0.893
Antibiotics	11.1 (22.2)	11.0 (19.6)	7.9 (16.4)	7.3 (31.4)	0.986	0.225	0.186	0.314	0.427	0.854
Depressive disease	136.4 (299.4)	101.9 (278.6)	67.2 (169.7)	38.4 (156.9)	0.501	**0.041**	** < 0.001**	0.358	**0.012**	0.088

*CS* Cushing’s syndrome, *ACS* Autonomous cortisol secretion, *NFAA* Non-functioning adrenocortical adenoma

*p* value assessed with independent samples *t* test

Thirteen patients died within a year from adrenalectomy (CS 5 patients, control group 8 patients). Median postoperative follow-up for drug consumption and ICD-diagnoses for the different tumour groups were: CS 3.0 (min–max 0–9.9) years, ACS 2.6 (0–9.6) years, and non-functioning adrenocortical adenoma 3.2 (0–9.9) years. Differences in drug consumption one year before and one year after adrenalectomy are presented in Table 4. Medication for hypertension increased at one year postoperatively in patients with CS and non-functioning adrenocortical adenoma. In patients with CS medication for hyperlipidaemia decreased after surgery and in patients with ACS there was a decrease in medication for depressive disorders as well.

**Table 4:** Differences in drug consumption one year before and one year after adrenalectomy for patients and controls with medication grouped according to anatomical therapeutic chemical (ATC) codes. Mean difference and standard deviation in defined daily dose (DDD) per patient per year is shown

	CS *n* = 127		ACS *n* = 45		NFAA *n* = 99		Control group *n* = 813	
	DDD, mean difference (SD)	*p* value	DDD, mean difference (SD)	*p* value	DDD, mean difference (SD)	*p* value	DDD, mean difference (SD)	*p* value
Hypertension	198.4 (728.1)	**0.003**	96.4 (598.4)	0.286	184.2 (510.0)	** < 0.001**	− 11.3 (364.2)	0.378
Diabetes	22.8 (184.7)	0.166	− 10.6 (164.7)	0.668	− 9.6 (193.9)	0.624	− 3.7 (90.4)	0.249
Hyperlipidaemia	− 59.4 (306.6)	**0.013**	81.6 (389.2)	0.167	29.2 (268.3)	0.281	− 3.7 (138.5)	0.450
Osteoporosis	− 14.3 (102.7)	0.119	7.5 (51.5)	0.336	5.7 (31.0)	0.072	− 2.1 (47.1)	0.198
Antibiotics	3.5 (47.9)	0.412	− 2.5 (22.5)	0.464	2.5 (19.8)	0.205	− 0.2 (20.6)	0.776
Depressive disease	− 30.3 (226.7)	0.134	− 47.6 (153.2)	**0.043**	4.1 (171.5)	0.814	− 1.0 (9.2)	0.784

Mean difference (SD), DDDpost-DDDpre

*CS* Cushing’s syndrome, *ACS* Autonomous cortisol secretion, *NFAA* Non-functioning adrenocortical adenoma

*p* value assessed with paired *t* test

**Fig. 2:**
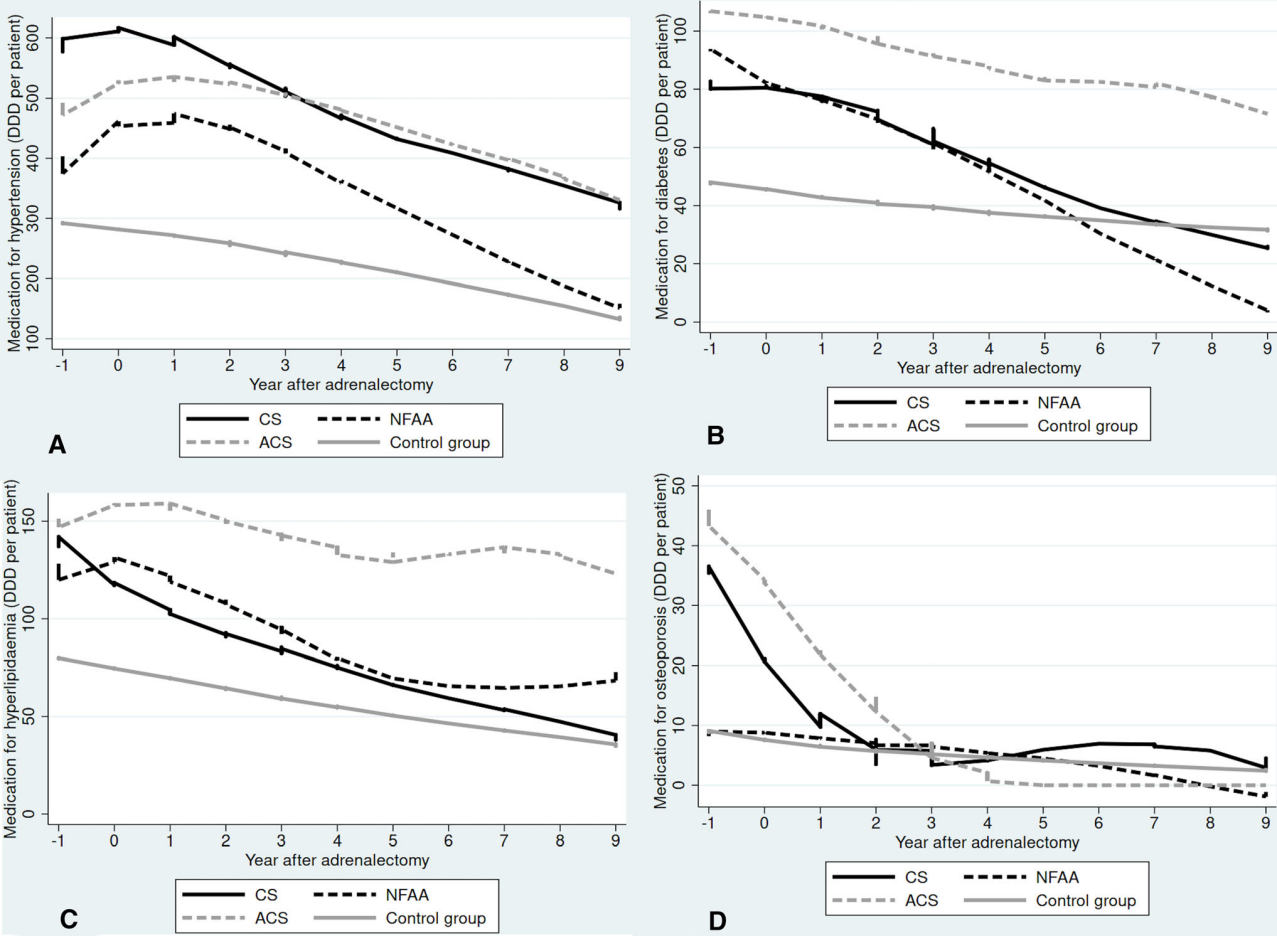
**a**–**d** Annual change in medication after adrenalectomy for hypertension, diabetes, hyperlipidaemia and osteoporosis. DDD (defined daily dose) per patient per year

The annual change in medication after adrenalectomy is presented in Fig. 2a–d. Medication for hypertension seems to increase immediately after adrenalectomy in all patient groups, followed by a distinct decrease over time. In patients with ACS, medication with antidiabetics and for hyperlipidaemia decreased only slightly during follow-up in contrast to the other patient groups where the levels decreased clearly over the years. Postoperatively the decrease in medication for osteoporosis in patients with CS and ACS was sharp over the first two years after surgery. Medication with antibiotics and antidepressants was fairly low and hence it is difficult to draw conclusions regarding change over time (Online Resource 1a–b).

**Fig. 3:**
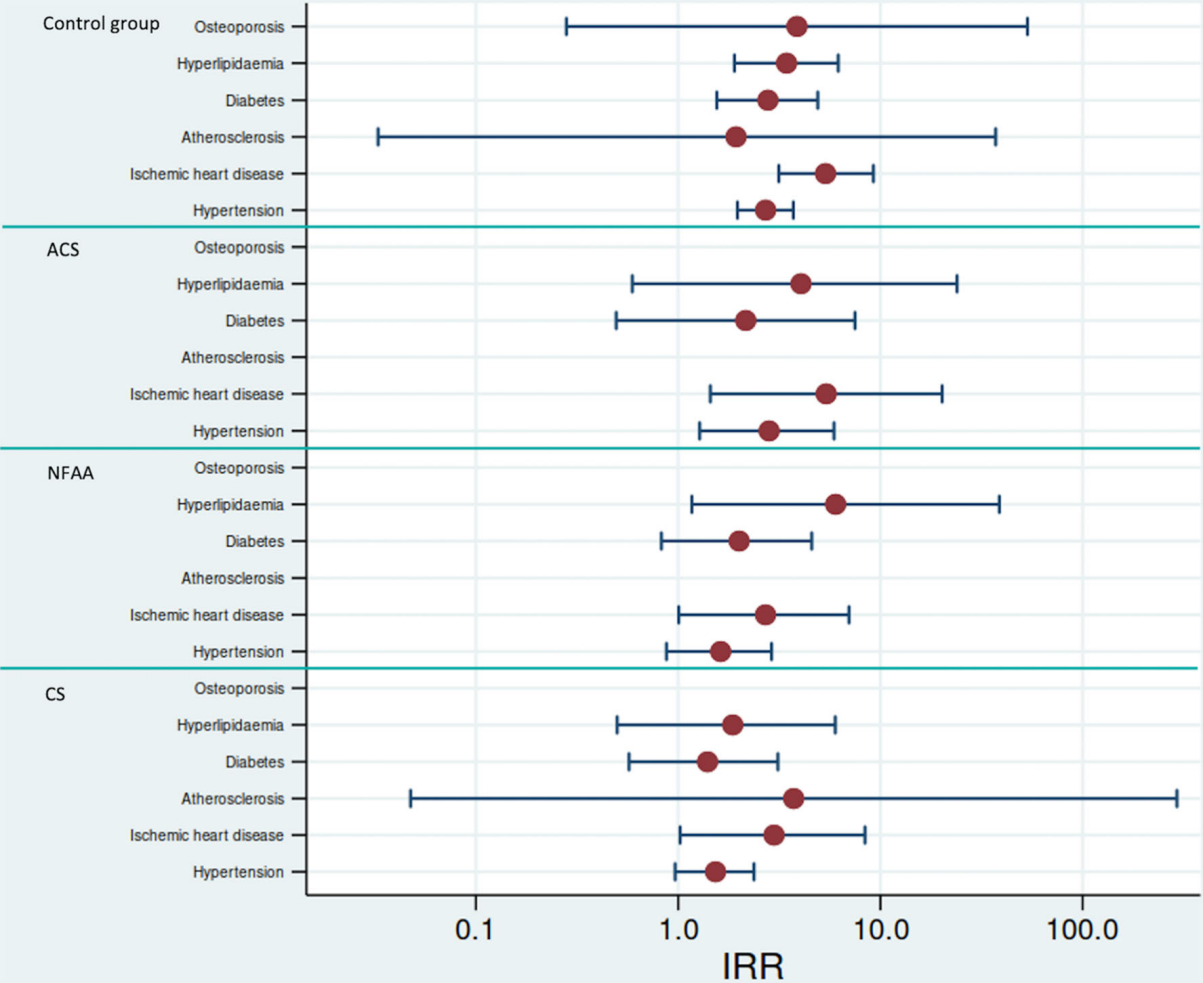
Morbidity in different disease groups calculated on inpatient diagnosis (ICD-10). Presented as density incidence rate ratios

Analysis of data from NPR in Sweden showed no large differences in morbidity among the patient groups. Incidence rate ratios increased after surgery, or the corresponding date in controls, for ischemic heart disease in all patient groups as well as in controls (Fig. 3).

## Discussion

Cushing’s syndrome is associated with high morbidity if untreated [27, 28]. This may also be the case in patients with ACS and so-called non-functioning adrenal adenoma. Results from mainly retrospective studies during the last 15 years show that patients with ACS have an increased risk for hypertension and type-2 diabetes, quite similar as for patients with CS [10, 11]. Hence, it has been suggested that adrenalectomy may ameliorate these cardiovascular risk factors in patients with ACS [9, 10, 12]. It has also been reported that patients with non-functioning adrenocortical adenoma have an increased risk for hypertension, type-2 diabetes and the metabolic syndrome [18, 19]. The effect of adrenalectomy is, however, unclear. In the context of the increasing diagnostic prevalence of adrenal incidentaloma, the effect of adrenalectomy on morbidity in these specific patient groups needs to be evaluated.

In the present study there were no major differences in morbidity, as evaluated with ATC-codes, for patients with CS and ACS before adrenalectomy. Nor for patients with ACS compared with non-functioning adrenocortical adenoma other than osteoporosis. Patient in all three groups used more antihypertensive drugs as compared to their controls, both before and after adrenalectomy. Medication increased the first year after adrenalectomy for all patient groups followed by a decrease annually. Distinct reductions annually were also detected for patients with CS for diabetes, hyperlipidaemia, osteoporosis, antibiotics and antidepressant medication. The results should be interpreted with some caution because of large statistic variability in the patient population and also because of possible reductions in medication for osteoporosis due to absence of fractures. The annual decrease in medication for diabetes and hyperlipidaemia were less distinct for patients with ACS compared with patients with CS. However, there were no differences in medication for hypertension or osteoporosis. Medication for all studied diseases decreased slightly over time including that of the control group. Possible causes include non-adherence, competing risks and secular trends. Interestingly, the frequency of hypertension and the use of antihypertensive drugs were higher for patients with non-functioning adrenocortical adenoma compared with controls, although lower than for patients with CS and ACS. Non-functioning adrenocortical adenoma are considered to compose no health risk, in this study they had an increased risk for antihypertensive medication. One reason for this could be to detection bias, although subtle hormonal hypersecretion not detected by current biochemical methods cannot be ruled out. Indeed, some reports indicate disturbances in cortisol secretion in patients with non-functioning adrenocortical adenoma compared with controls [29]. Unfortunately, exact ACTH and cortisol value after DST is lacking in the database and inadequate testing could not be ruled out.

Hypertension is the most frequent treatable risk factor for cardiovascular disease with prevalence in European countries of 25–44 percent [30, 31]. In the present study it was near 50 per cent in all tumour groups. Interestingly, the frequency of hypertension in non-functioning adrenocortical adenoma has been reported to be 72.5–75.3 percent [18, 21].

Most studies agree that non-functioning adrenocortical adenoma and ACS rarely progress to CS [3, 7, 32]. Patients with ACS have a distinct steroid fingerprint that separates them from patients with CS [33]. Papanastasiou et al. found that 31 percent of patients initially diagnosed with non-functioning adrenocortical adenoma developed ACS over 5.5 years [34]. Hence, non-functioning adrenocortical adenoma and ACS might be part of a biochemical spectrum, and if so, the DST might not be a reliable predictor for metabolic alterations in patients with non-functional adrenal incidentaloma. Based on current guidelines in healthy patients with lipid rich, small non-functioning adrenocortical adenoma further follow-up is not warranted [16].

Despite many reports of increased prevalence of cardiovascular risk factors in patients with non-functioning adrenocortical adenoma, there is a lack of data about the effects of adrenalectomy [35]. It may be hypothesized that this group of patients has a greater burden of medical problems and therefore is more likely to undergo abdominal imaging. The results from the present study are generally in agreement with results from previous reports showing that patients with non-functioning adrenocortical adenoma have an increased risk for hypertension and diabetes mellitus compared with healthy controls [19–21]. In this study a decrease in drug consumption for hypertension, diabetes and hyperlipidaemia was observed after adrenalectomy. However, a recent study of health related quality of life showed that patients with non-functioning adrenocortical adenoma did not benefit from adrenalectomy [36].

Evaluation of drug use indicated a higher risk for hypertension, hyperlipidaemia, diabetes, osteoporosis and depression in patients with ACS compared with healthy controls but improvements could not be verified postoperatively neither in drug consumption, nor hospital care as assessed by in-patient ICD-10-codes. Current evidence does not allow general treatment guidelines in patients with ACS, individual evaluation and care are recommended [16, 37]. The results in the present investigation show that caution is needed when interpreting any impact of adrenalectomy on ACS in observational studies [8]. Further information will hopefully be coming from the European multicentre randomized controlled trial (NCT01246739 [38]).

The accuracy of drug-based morbidity derived from ATC-codes has been debated. ATC-codes used in this study are comparable with earlier evaluations [39]. A number of limitations of the current study are acknowledged. The study was based on results from a database with predefined data fields with some important biochemical variables missing. Observational studies can suffer from confounding and selection-bias. Diagnostic criteria for hypercortisolism have changed over time and may differ between clinics. Further, morbidity was assessed by inpatient-ICD-diagnoses. Since patients often are managed in primary care for some of the studied diseases, missing new cases and overestimation by pre-existing disease could hamper estimation of morbidity. However, the use of prescribed drugs would capture the morbidity of all patients and should complement the inpatient-diagnoses. The strength of the present study is that data was extracted from controlled and mandatory national registries and with data from SQRTPA, which was cross-linked with data from the SPDR.

The risk for hypertension for patients with CS, ACS and non-functioning adrenocortical adenoma were high in this register-study. After the initial postoperative year an annual decrease in medication with antihypertensive drugs was noted for these patients. Adrenalectomy failed to decrease morbidity in cardiovascular disease based on inpatient-diagnosis in a short perspective. A prospective, randomized trial is hence warranted to evaluate the long-term impact of adrenalectomy on morbidity in patients with ACS and in patients with non-functioning adrenocortical adenoma.

## Supplementary Information

Below is the link to the electronic supplementary material.

Supplementary file1 (PDF 183 kb)

## Declaration

All authors comply with the journal’s policies.
