# Supplementary material for: Impact of Adrenalectomy on Morbidity in Patients with Non-Functioning Adrenal Cortical Tumours, Mild Hypercortisolism and Cushing’s Syndrome as Assessed by National and Quality Registries
Source: World J Surg. 2021 Jun 27;45(10):3099–107. doi: 10.1007/s00268-021-06214-0 (PMC8408086; doi:10.1007/s00268-021-06214-0)
Supplement: Supplementary file 1 — Supplementary file1 (PDF 183 kb) [file 268_2021_6214_MOESM1_ESM.pdf]

Title:

Impact of adrenalectomy on morbidity in patients with non-functioning adrenal cortical tumours, mild hypercortisolism and Cushing's syndrome as assessed by national and quality registries.

Authors:

Lo Hallin Thompson[1, 2]

Jonas Ranstam [2, 3]

Martin Almquist[1, 2]

Erik Nordenström[1, 2]

Anders Bergenfelz[1, 2]

Affiliations and addresses:

1 Department of Surgery, Skåne University Hospital, 22185 Lund

2 Department of Clinical Sciences, Lund University, Lund, Sweden

3 Department of Orthopaedics, Skåne University Hospital, 22185 Lund

Corresponding author:

E-mail: lo.hallin-thompson@med.lu.se

Address: Skånes Universitetssjukhus, 22185 Lund, Sweden

Tel: +46708218513

Fax: +4646172335

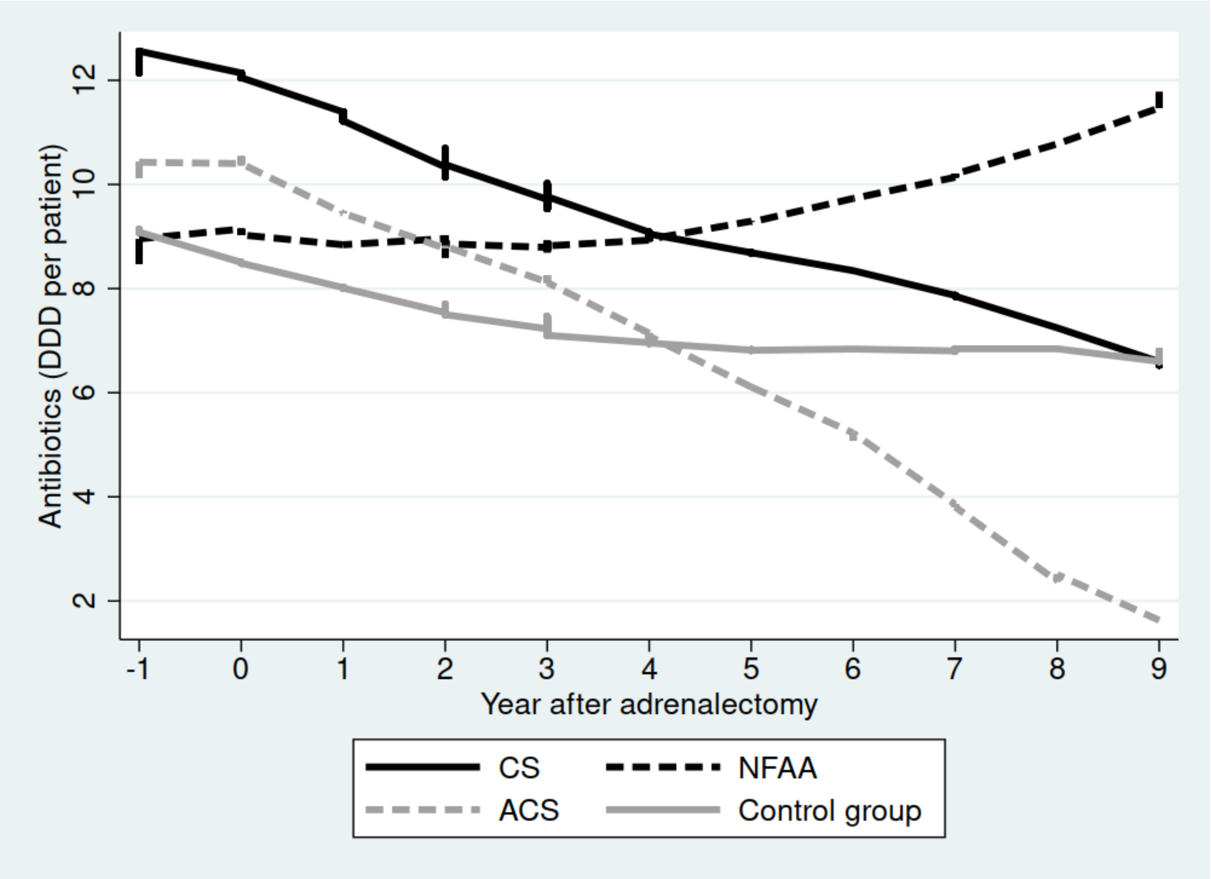

Online Resource 1a Annual change in medication with antibiotics, DDD (defined daily dose) per patient per year

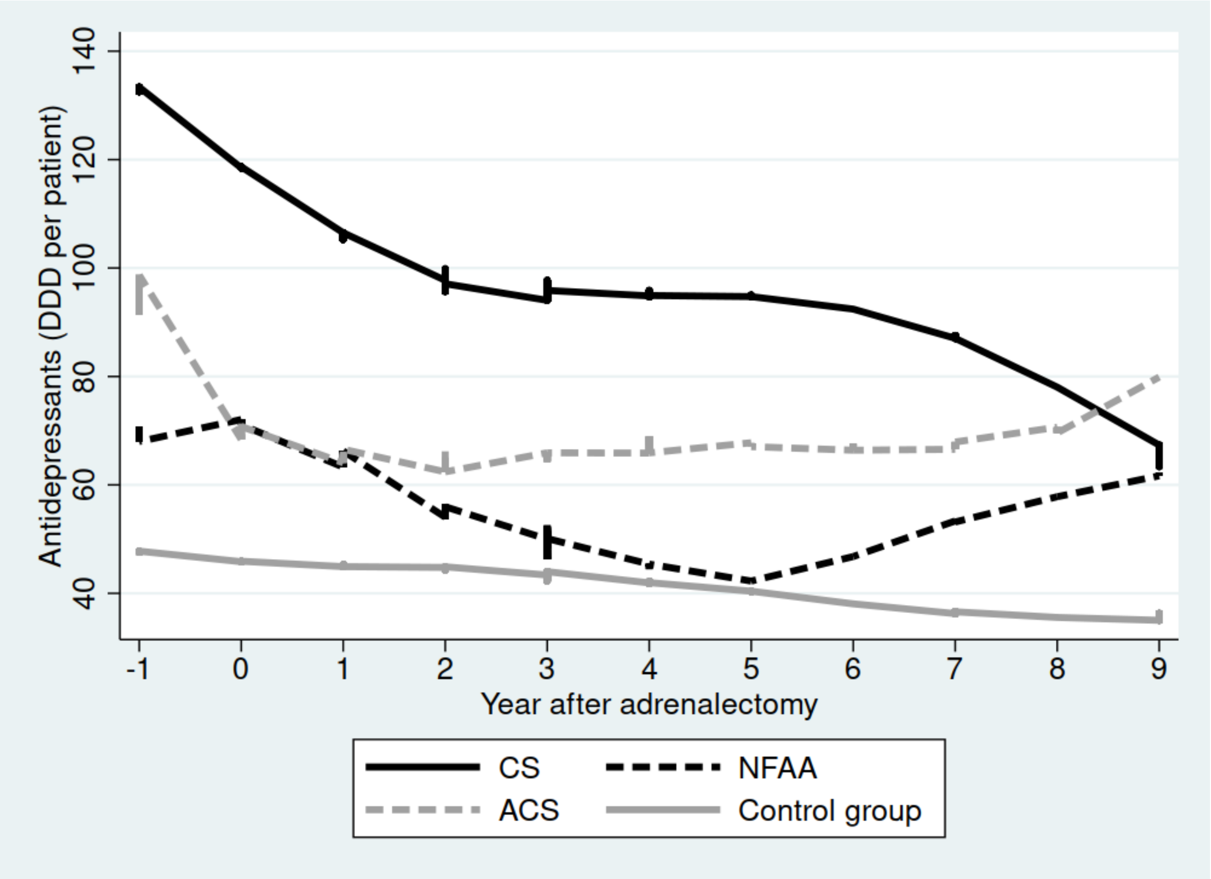

Online Resource 1b Annual change in medication with antidepressants, DDD (defined daily dose) per patient per year
